# Hysterosalpingography and Ultrasonography Features of Herlyn-Werner-Wunderlich Syndrome Detected during Infertility Workup

**DOI:** 10.1155/2024/1055130

**Published:** 2024-02-19

**Authors:** Hidayatullah Hamidi, Bibi Hosai Balkhi

**Affiliations:** ^1^Radiology Department, French Medical Institute for Mothers and Children (FMIC), Kabul, Afghanistan; ^2^Dawat University, Kabul, Afghanistan

## Abstract

The Herlyn-Werner-Wunderlich syndrome (HWWS) is a very rare congenital anomaly of the urogenital tract. It is characterized by a combination of didelphys uterus, unilateral vaginal obstruction, and ipsilateral renal agenesis. MRI imaging is usually used for diagnosis; however, the authors present a case of HWWS diagnosed by ultrasonography (HSG) and hysterosalpingography (HSG) in a 22-year-old lady who has undergone an imaging workup of infertility.

## 1. Background

The Herlyn-Werner-Wunderlich syndrome (HWWS) also known as uterus didelphys with obstructed hemivagina and ipsilateral renal agenesis (OHVIRA) syndrome is a rare congenital anomaly of the urogenital tract [1]. As the development of reproductive system is near the urinary tract and kidneys having some common embryonic structures, this anomaly of uterus is almost always associated with ipsilateral renal agenesis [2]. This pathology is well known, and the imaging features are properly described in the literature, but what this case brings new is the hysterosalpingography features of this entity which is not described in the literature.

## 2. Case Presentation

A 22-year-old married lady with chief complaint of infertility for 3 years is undergoing imaging workup. She also complains from chronic pelvic pain especially during and soon after the menstrual cycle. No current or previous sign and symptom related to the lower urinary tract were documented.

**Figure 1:**
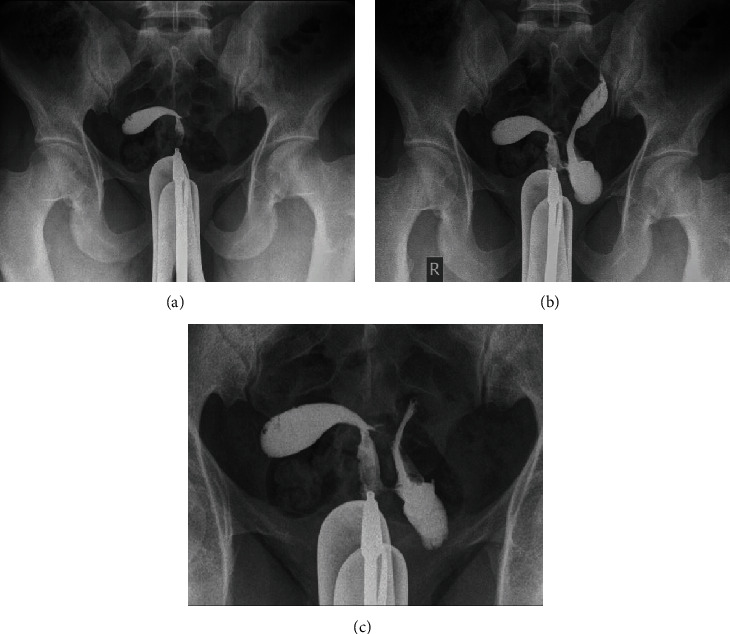
(a) Frontal radiographic just after contrast injection through vaginal cannula: opacification of tubular shape of the endometrial cavity to the right side of the pelvis raising the possibility of unicornuate uterus. (b) Frontal radiographic after few minutes of contrast injection through vaginal cannula: opacification of a tract to towards left side likely at lower cervix or upper vagina and subsequent opacification of second endometrial cavity in the left side. There is also opacification of left hemivagina caudally which is distended due to blind end. (c) Delayed frontal radiograph for evaluation of fallopian tubes. No opacification of fallopian tube is noted in either side.

She was prescribed hysterosalpingography (HSG). The exam was performed under fluoroscopy in aseptic condition. In the early image, a tubular shape of the endometrial cavity was opacified in the right side which was raising the possibility of unicornuate uterus (Figure 1(a)); however, at later images, there was a tract going to the left side of the midline with subsequent opacification of the second endometrial canal cranially and the left hemivagina caudally (Figure 1(b)). Nonopacification of the fallopian tubes was seen in either side at delayed images (Figure 1(c)) suggesting bilateral blocked fallopian tubes.

These findings concluded double vaginal cavities with bicornuate bicollis uterus (didelphys), obstructed left hemivagina, and communication between them at lower cervix/upper vaginal level.

**Figure 2:**
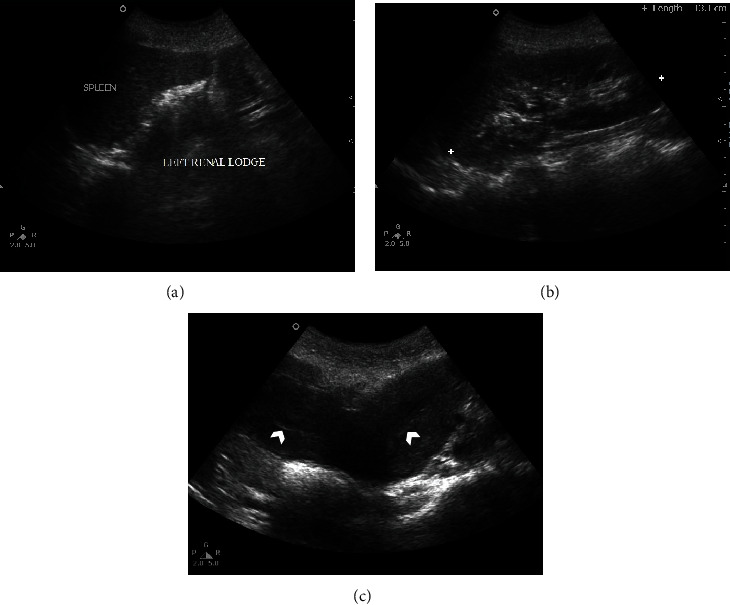
(a) Gray-scale ultrasound image of left renal lodge with nonvisualization of the left kidney. (b) Gray-scale ultrasound image of right renal lodge showing relatively compensatory prominent size of right kidney. (c) Gray-scale ultrasound image of the pelvis showing both uterine bodies. No dilatation of the endometrial canals (white arrowheads).

Considering these findings, a complementary ultrasonography (USG) was performed for the patient to look for the kidneys, which showed an absent left kidney and compensatory prominent size of right kidney (Figures 2(a) and 2(b)). Gray-scale USG image of the pelvis depicted both uterine bodies. No dilatation of the endometrial canals was present (Figure 2(c)).

**Figure 3:**
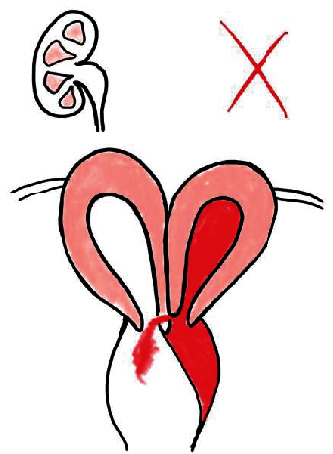
Schematic representation of the patient's pathology: uterus didelphis with obstructed left hemivagina and ipsilateral renal agenesis.

The overall findings were typical for uterus didelphys with obstructed hemivagina and ipsilateral renal agenesis, also known as the Herlyn-Werner-Wunderlich syndrome (Figure 3).

As the patient was only referred for imaging workup to the authors' department, no further details about management and follow-up are available.

## 3. Discussion

HWW syndrome is a rare female urogenital anomaly that can present various combinations of uterine anomalies, unilateral cervicovaginal obstruction, and ipsilateral renal anomalies [3].

HWW syndrome represents a type of Müllerian duct anomalies (MDA) associated with mesonephric duct anomalies. The incidence of didelphys uterus, related to HWWS, is reported about 1/2,000 to 1/28,000 and associated with unilateral renal agenesis in 43% of cases. Vaginal septum is reported present in about 75% of women with didelphys uterus [1].

With normal external genitalia, HWWS is usually asymptomatic until menarche when patients present with worsening abdominal pain during menses and a palpable pelvic or abdominal mass [4].

**Figure 4:**
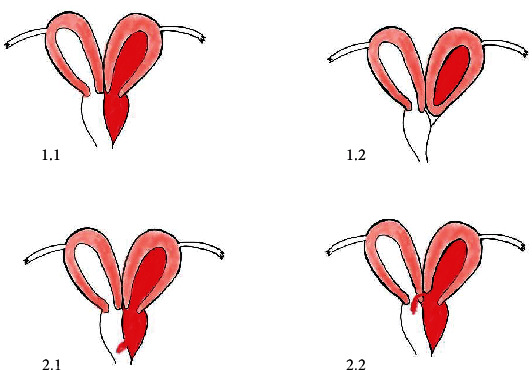
Schematic representation of classification of HWWS.

The entity is classified into two main types that are further categorized into two subtypes (Figure 4):

(I) Completely obstructed hemivagina:
  1. With blind hemivagina
  2. Cervicovaginal atresia without communicating uteri
(II) Incompletely obstructed hemivagina:
  1. Partial reabsorption of the vaginal septum
  2. With communicating uteri [4]

### 3.1. Clinical Perspective

Clinical presentation in HWWS depends on classification.

Dysmenorrhea, abdominal pain, fever, vomiting, and endometriosis are common in type I, and haematometra, haematosalpinx, and haematoperitoneum are also very common. Irregular per vaginal bleeding, intermittent mucopurulent discharge, and pelvic inflammatory disease are less common in type I.

On the other hand, irregular per vaginal bleeding and intermittent mucopurulent discharge and pelvic inflammatory disease are more common in type II. Abdominal pain, fever, vomiting, haematometra, haematosalpinx, haematoperitoneum, and endometriosis, however, are uncommon in type II.

Prognosis to secondary endometriosis, pelvic adhesions, pyosalpinx, and pyocolpos are quick in type I, but it gradually occurs in type II [5].

### 3.2. Imaging Perspective

The diagnosis of HWWS relies on radiologic findings [6]. Imaging modalities delineate the anatomic variation of the genitourinary tract: a didelphys uterus, unilateral vaginal obstruction, and ipsilateral renal agenesis. MRI and transvaginal USG can depict these defects. The transvaginal ultrasonography, an easily accessible and cheap option, provides good imaging of the uterus and adnexa without any radiation exposure. If there is hematocolpos, USG may show it as a hypoechoic mass between the bladder and the rectum [7]; however, the presence of hematocolpos may distort the anatomy and make the diagnosis challenging [8].

MRI can evaluate uterine morphology, detect communication between vaginal and uterine lumens, characterize fluid contents, and diagnose the complications like endometriosis [9].

HSG, the conventional imaging modality used for anatomic evaluation of uterus and patency of fallopian tubes, can be helpful in the diagnosis of classifications II (1) and II (2) where there is a communication between both side, while it can give incorrect imaging of the anatomy in classifications I (1) and I (2) which will appear as unicornuate unicollis uterus on HSG.

Ultrasound-guided endoscopy can also play important role in the evaluation of complex anomalies [10].

Imaging features of similar case (classification II (2)) are reported from the same department previously [7], but what makes this case different from the published ones is the focus on hysterosalpingography features of this entity.

### 3.3. Treatment and Prognosis

Treatment for patients with classification I (2) is different from patients with other classifications. As it is difficult to correct cervical agenesis surgically, laparoscopic or transabdominal resection of the affected ipsilateral uterus is suggested [4]. Resection of the vaginal septum is the treatment of choice for obstructed hemivagina with hematocolpos [11]. Vaginal septotomy is preferred to be done by hysteroscopy rather than laparoscopic technique [9].

## 4. Take Home Message/Conclusion

HWWS can sometimes be diagnosed by relatively simple imaging techniques; hence, the ultrasonologist, radiologist, gynecologist, and urologist should be prepared to see uncommon and complex entities during daily practice.

## Data Availability

The data will be available for the journal editor as per request.

## Abbreviations

HWWS: Herlyn-Werner-Wunderlich syndrome
OHVIRA: Obstructed hemivagina and ipsilateral renal agenesis
MDA: Müllerian duct anomalies
MRI: Magnetic resonance imaging
USG: Ultrasonography.
